# Impact of school-based vegetable garden and physical activity coordinated health interventions on weight status and weight-related behaviors of ethnically diverse, low-income students: Study design and baseline data of the Texas, Grow! Eat! Go! (TGEG) cluster-randomized controlled trial

**DOI:** 10.1186/s12889-016-3453-7

**Published:** 2016-09-13

**Authors:** A. Evans, N Ranjit, D. Hoelscher, C. Jovanovic, M. Lopez, A. McIntosh, M. Ory, L. Whittlesey, L. McKyer, A. Kirk, C. Smith, C. Walton, N. I. Heredia, J. Warren

**Affiliations:** 1 Michael & Susan Dell Center for Healthy Living - Division of Health Promotion and Behavioral Sciences - University of Texas Health (UTHealth) Science Center, Austin Regional Campus, Austin, USA; 2 Division of Behavioral Science and Health Promotion, University of Texas Health Science Center (UTHealth) School of Public Health, Houston, USA; 3 Family Development & Resource Management, Texas A&M AgriLife Extension Service, College Station, USA; 4 Recreation, Park and Tourism Sciences & Sociology, Texas A&M University, College Station, USA; 5 Health Promotion and Community Health Sciences, Texas A&M Health Science Center School of Public Health, College Station, USA; 6 Department of Horticultural Sciences, Texas A&M AgriLife Extension Service, College Station, USA; 7 College of Education and Human Development, Transdisciplinary Center for Health Equity Research, Texas A&M University, College Station, USA; 8 Center for Health Promotion and Prevention Research, University of Texas Health Science Center (UTHealth) School of Public Health, Houston, USA

**Keywords:** School garden intervention, Physical activity intervention, JMG, LGEG, WAT, Randomized controlled trial, Low-income children, Hispanic, African American

## Abstract

**Background:**

Coordinated, multi-component school-based interventions can improve health behaviors in children, as well as parents, and impact the weight status of students. By leveraging a unique collaboration between Texas AgriLife Extension (a federal, state and county funded educational outreach organization) and the University of Texas School of Public Health, the Texas Grow! Eat! Go! Study (TGEG) modeled the effectiveness of utilizing existing programs and volunteer infrastructure to disseminate an enhanced Coordinated School Health program. The five-year TGEG study was developed to assess the independent and combined impact of gardening, nutrition and physical activity intervention(s) on the prevalence of healthy eating, physical activity and weight status among low-income elementary students. The purpose of this paper is to report on study design, baseline characteristics, intervention approaches, data collection and baseline data.

**Methods:**

The study design for the TGEG study consisted of a factorial group randomized controlled trial (RCT) in which 28 schools were randomly assigned to one of 4 treatment groups: (1) Coordinated Approach to Child Health (CATCH) only (Comparison), (2) CATCH plus school garden intervention [Learn, Grow, Eat & Go! (LGEG)], (3) CATCH plus physical activity intervention [Walk Across Texas (WAT)], and (4) CATCH plus LGEG plus WAT (Combined). The outcome variables include student’s weight status, vegetable and sugar sweetened beverage consumption, physical activity, and sedentary behavior. Parents were assessed for home environmental variables including availability of certain foods, social support of student health behaviors, parent engagement and behavior modeling.

**Results:**

Descriptive data are presented for students (*n* = 1369) and parents (*n* = 1206) at baseline. The sample consisted primarily of Hispanic and African American (53 % and 18 %, respectively) and low-income (i.e., 78 % eligible for Free and Reduced Price School Meals program and 43 % food insecure) students. On average, students did not meet national guidelines for vegetable consumption or physical activity. At baseline, no statistical differences for demographic or key outcome variables among the 4 treatment groups were observed.

**Conclusions:**

The TGEG study targets a population of students and parents at high risk of obesity and related chronic conditions, utilizing a novel and collaborative approach to program formulation and delivery, and a rigorous, randomized study design.

## Background

Although some leveling of the increase in incidence of childhood obesity has been noted, childhood obesity continues to be an ongoing problem in the United States (U.S.) [1]. In 2011–2012, 34 % of children ages 6 to 11 were overweight or obese, and 18 % were obese. Among Hispanic children of the same age, these figures were 46 % and 26 %, respectively [1]. Specific to Texas, Hispanic child (ages 2–19) obesity rates range from 20 % - 30 % [2].

Several behaviors have been shown to impact students’ weight status, including fruit and vegetable consumption [3, 4], sugar sweetened beverage (SSB) consumption [5, 6], engagement physical activity (PA) [7, 8] and sedentary behavior [6, 9]. Because parents are the main gatekeepers to younger children’s dietary and PA behaviors, several parental behaviors are also important for maintaining and decreasing a student’s weight status, including increasing access and availability of vegetables at home [10–13], limiting availability of SSB at home [11, 14] providing social support for PA [15, 16], limiting student’s sedentary activity [17], preparing food together [18] and eating meals together with their children [19–22], and doing PA with their children [23].

School interventions can play an important role in the prevention of childhood obesity [24–27]. Schools are uniquely positioned to have a positive impact on students’ knowledge and behaviors related to nutrition and PA by creating a healthy environment. In addition, schools can provide an effective way to reach parents, who are otherwise often difficult to reach. Although school-based nutrition and PA interventions have shown significant effects on students’ behaviors, few school-based interventions have incorporated multiple strategies such as gardening, nutrition, and PA components into one intervention.

School-based interventions using gardening as a key component are a promising approach to addressing healthy eating and student’s weight status. Recent studies of garden-based approaches in schools show successful engagement of students and parents, including minority students [28] and students living in limited resource households [29]. Garden-based interventions consistently demonstrate their ability to increase knowledge and preference for vegetables among students. However, evidence indicating positive impacts on actual dietary behaviors and child weight status is mixed [30–42]. Only one study has found a significant reduction in body mass index (BMI) following a gardening intervention [42]. Therefore, further research using large-scale studies is needed to examine if garden-based programs can effectively impact students’ BMI levels.

School-based PA interventions are one method to increase children’s PA levels [43–46]. In addition to the more traditional interventions that focus on changing the curriculum, PA interventions focusing on non-curricular activities such as classroom breaks, system-wide school changes and family components can also be impactful [47]. By focusing on non-curricular strategies, these types of interventions can help address the common barriers to school-based PA interventions, which include lack of time during school hours due to the need to teach standardized test focused lessons [48, 49]. However, in regard to reducing BMI, results of these types of PA programs are mixed. Further research must be conducted to determine the impact of non-curricular PA interventions on students’ behavior and weight status.

A combination of nutrition, gardening, and PA interventions in schools can theoretically work synergistically to improve health behaviors in students and weight status of students [50, 51]. However, there is a gap in the literature with regard to the impact of such combined interventions, because they are logistically difficult to implement, involving expertise in a variety of specialties; are very resource intensive; and because people still tend to think in intervention silos. To address this gap, the Texas Grow! Eat! Go! (TGEG) study was developed to assess the independent and combined impact of gardening, nutrition and PA intervention(s) on the prevalence of healthy eating and PA behaviors and weight status among low-income elementary students and parents. In particular, TGEG pioneered the collaboration between existing Extension resources and evidence-based Coordinated School Health (CSH) programming to deliver a uniquely comprehensive and coordinated intervention. Using a factorial group randomized controlled trial (RCT) with 28 low-income elementary schools, effects of the different combination of interventions were evaluated. The purpose of this paper is twofold: 1) to describe the intervention protocol, research design, and details of the data collection protocol used in the TGEG Study and 2) to present selected baseline data for participating students and parents.

## Methods

### Study design

**Fig. 1:**
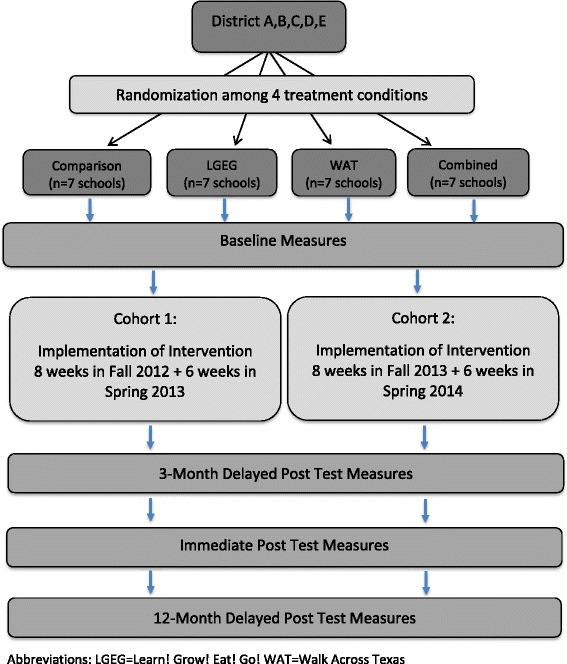
Study design

The TGEG intervention study was funded for a 5-year period starting in 2011. The study design consisted of a factorial group RCT in which 28 schools from geographically separate areas of Texas were randomly assigned to one of four treatment groups (Fig. 1). In Texas, all elementary schools are required by state policy to implement a specific Texas Education Agency-approved CSH program (TEC §38.0141). To ensure comparability of the participating study schools, the researchers recruited schools that had selected the Coordinated Approach to School Health (CATCH) program (described more fully in the Methods section) as their CSH program [52]. Accordingly, the four treatment groups included (1) CATCH only (*Comparison*), (2) CATCH plus a school garden intervention (*Learn, Grow, Eat & Go!* or *LGEG*), (3) CATCH plus a PA program (*Walk across Texas* or *WAT*), and (4) CATCH plus LGEG plus WAT (*Combined*).

The goal of the TGEG study was to measure the impact of the different combinations of interventions on key outcome variables related to healthy eating, PA and student weight status among 3rd grade students and their parents. Our hypotheses stated that students and parents in the combined treatment group would score higher on key outcome variables compared to students in the single intervention groups and the comparison group. A split cohort design was used to implement the study (i.e. cohort 1 began in the 2012 school year and cohort 2 in the 2013 school year). Data collection for cohort 1 occurred during the fall of 2012, spring and fall of 2013 and spring 2014. For cohort 2, data collection occurred fall 2013, spring and fall 2015 and spring 2015. The TGEG pilot study, which was conducted in 2011–2012, assessed project feasibility and provided data on best practices for combining the multiple interventions, on appropriate implementation and data collecting practices, and on appropriateness of the data collection tools [53].

The key student outcome variables included objectively-measured BMI, and self-reported variables including (1) vegetable preference, (2) vegetable exposure, (3) vegetable consumption, (4) SSB consumption, (5) moderate and vigorous physical activity (MVPA), and (7) sedentary behaviors. The key parent outcome variables included (1) home availability/accessibility of vegetables, (2) home availability of SSB, (3) parental emotional social support for increasing PA, and (4) parental support for decreasing sedentary behavior. Lastly, the key student-parent interaction variables included (1) gardening together, (2) eating meals together, (3) engaging in PA together and (4) preparing food together. Table 1 presents summary information on the self-reported key outcome variables.

**Table 1:** Key outcome variables of TGEG project

Outcome variable	Example item	# Items	Response Options	Mean (SD)	Actual range	Cronbach’s alpha	Spear-man’s rho
Student							
Vegetables preference	Do you like to eat…? (list of 19 vegetables)	19	0–1 (0 = No, 1 = Yes)	8.9 (4.1)	0–19	0.8	
Vegetable Exposure	Have you eaten…? (list of 19 vegetables)	19	0–1 (0 = No, 1 = Yes)	12.2 (4.0)	0–19	0.8	
Vegetable consumption	Yesterday, did you eat any orange vegetables like carrots, squash, or sweet potatoes?	3	0–3 (0 = None, 1 = 1 time yesterday, 2 = 2 times, 3 = 3 or more times yesterday)	2.63 (2.5)	0–9	0.7	
SSB consumption	Yesterday, did you drink any regular sodas or soft drinks?	2	0–3 (0 = None, 1 = 1 time yesterday, 2 = 2 times, 3 = 3 or more times yesterday)	2.26 (1.8)	0–6		0.3
MVPA	Yesterday, did you do any moderate or vigorous physical activities for about 30 min (e.g., the time it takes to watch a cartoon) throughout the day? (Count in school and out of school activities.)	1	0–1 (0 = No, 1 = Yes)	88 % “yes” (0.9)	0–1	NA	
Sedentary behavior	Yesterday, how many hours did you sit playing on the computer away from school?	3	0–4 (0 = No sedentary time, 1 = Less than 1 h, 2 = more than 1 but less than 2 h, 3 = more than 2 but less than 3 h, 4 = more than 3 h)	56.0 % >2 h in sed. beh.	0–12	0.6	
Parent							
Home availability vegetables	Last week, did you have…cut-up fresh vegetables/salad in your home?	5	0–3 (0 = Never, 1 = Some of the time, 2 = Most of the time, 3 = All of the time)	8.62 (3.1)	0–15	0.7	
Home availability SSB	Last week, did you have…soft drinks or sugar-sweetened beverages in your home?	1	0–3 (0 = Never, 1 = Some of the time, 2 = Most of the time, 3 = All of the time)	1.59 (0.9)	0–3	NA	
Parental emotional support for increasing PA	I encourage my child to play sports or do physical activities.	5	0–4 (0 = Strongly disagree, 1 = Disagree, 2 = Neither agree nor disagree, 3 = Agree, 4 = Strongly agree)	15.12 (4.4)	0–20	0.8	
Parental support for decreasing sedentary behavior	I show approval when my child is physically active.	3	0–4 (0 = Strongly disagree, 1 = Disagree, 2 = Neither agree nor disagree, 3 = Agree, 4 = Strongly agree)	6.38 (2.7)	0–12	0.6	
Student/Parent Interaction							
Gardening together	During the last school year have you done any of the following at school OR home: Weeded or waters a garden with your child(ren)?	5	0–2 (0 = Never, 1 = Once, 2 = More than once)	2.14 (1.9)	0–5	0.8	
Eating meals together	During the week, did you do the following with your child? Ate evening meal together.	2	0–2 (0 = Never or almost never, 1 = sometimes, 2 = Almost always or always)	2.72 (1.2)	0–4		0.6
Engaging in PA together	During the last week, how many days were you physically active with your child, not including walking (for example, swimming, jogging, playing basketball or soccer, etc.)?	2	0–4 (0 = Strongly disagree, 1 = Disagree, 2 = Neither agree nor disagree, 3 = Agree, 4 = Strongly agree)	3.81 (2.6)	0–8		0.6
Preparing food together	During the week, did you do the following with your child? Prepared food together.	2	0–1 (0 = No, 1 = Yes)	1.28 (0.8)	0–2		0.4

## Intervention overview and description

### Intervention selection and theoretical framework

Two previously developed Texas A&M AgriLife Extension programs (i.e., *Junior Master Gardener Health and Nutrition from the Garden* and the *Walk Across Texas* programs) were adapted to create the garden and PA interventions for this study. The overarching goals for both interventions were to engage children both at school and at home. Social Cognitive Theory (SCT) served as the framework for the development of the specific strategies included in the interventions. SCT posits that behavior is influenced by individual and environmental factors. In addition, it provides specific strategies to increase desired behaviors. For example, self-efficacy, a key construct in SCT, can be enhanced by skill building and positive reinforcement [54]. Table 2 provides information on specific intervention components.

**Table 2:** Intervention components implemented for the TGEG Study using the Behavior Change Technique Taxonomy [74]

Intervention	Intervention components	Target	Behavior change techniques	Implementation agent
LGEG	LGEG Training	Teachers	Instructions on how to perform a behavior (4.1); Anticipation of future reward (14.10); Identification of self as role model (13.1)	TGEG team, teachers, project specialists
	School garden growing 12 vegetables (bell pepper, bok choy, broccoli, carrots, cherry tomatoes, cauliflower, potatoes, red leaf lettuce, spinach, squash, sugar snap peas, Swiss chard)	Students	Vicarious reinforcement (16.3); Instructions on how to perform a behavior (4.1); Anticipation of future reward (14.10);	Teachers, volunteers, Extension agents
	14 horticulture & nutrition science classroom lessons related to what plants need to grow, what our bodies need to grow, and integration of gardening and nutrition within core subject areas	Students	Behavioral experiments (4.4); Instructions on how to perform a behavior (4.1)	Teacher
	12 Classroom vegetable recipe demo & tastings * 12 recipes in English/Spanish	Students Parents	Behavioral practice (8.1); Social consequences (5.3); Behavior substitution (8.2)	Extension agents, volunteers and project specialists
	Student journal in which student completes activities related to nutrition, vegetable tasting, garden experiences, classroom science, math, and language arts learning objectives	Students	Goal setting (1.1); Identification of self as role model (13.1)	Teachers
	LGEG website web videos of gardening instruction, harvest guidance, vegetable preparation by kids, vegetable variety/growing chart by region	Teachers Students Parents	Vicarious reinforcement (16.3); Instructions on how to perform a behavior (4.1); Anticipation of future reward (14.10);	TGEG team
	"Dinner Tonight" web videos of adults preparing recipes	Parents	Vicarious reinforcement (16.3); Instructions on how to perform a behavior (4.1); Anticipation of future reward (14.10); Restructuring of physical and social environment (12.1 and 12.1)	Extension agents
	14 Take-home family stories promoting healthy meals, water consumption, walking & outdoor play, and container gardening	Parents, Students	Instructions on how to perform a behavior (4.1); Anticipation of future reward (14.10); Restructuring of physical and social environment (12.1 and 12.1)	Teachers
	LGEG Toolkit - materials, supplies, classroom children's books	Teachers school staff	Modeling of behaviors (6.1); Goal setting (1.1); Review of outcome goals (1.7)	Teachers
WAT	WAT Training	Teachers	Instructions on how to perform a behavior (4.1); Anticipation of future reward (14.10); Identification of self as role model (13.1)	TGEG team, teachers, project specialists
	WAT kick-off Event /Celebration	Teachers Parents, Students	Restructuring of physical and social environment (12.1 and 12.1); Identification of self as role model (13.1)	Teachers and extension agents
	8 week Classroom Competition	Teachers Parents, Students	Restructuring of physical and social environment (12.1 and 12.1); Identification of self as role model (13.1); Social reward (10.4)	Teachers and extension agents
	3^rd^ Grade Teacher Lesson Plans – 30 Classroom Activity Breaks by subject matter/learning objectives –math, science, language arts, health	Students	Restructuring of physical and social environment (12.1 and 12.1);	Teachers
	10 Parent – Newsletters (English/Spanish), Walking Bingo Card, Bonus Miles Record (English/Spanish)	Parents, Students	Instructions on how to perform a behavior (4.1); Anticipation of future reward (14.10); Identification of self as role model (13.1); Social support (3.2 and 3.3)	Teachers
	Before / After School Extracurricular Activities Related to Physical Activity (i.e. running clubs)	Students	Instructions on how to perform a behavior (4.1); Anticipation of future reward (14.10); Social support (3.2 and 3.3)	Teachers, volunteers, project specialists
	Walk Across Texas Website: Teacher guide, registration forms, mileage calculator, mileage record, certificates	Teachers Parents, Students	Modeling of behaviors (6.1); Goal setting (1.1); Review of outcome goals (1.7)	TGEG team
	WAT Toolkit - materials, supplies, classroom children's books	Teachers school staff	Modeling of behaviors (6.1); Goal setting (1.1); Review of outcome goals (1.7)	Teachers
CATCH	CATCH Training	Teachers/ School staff	Instructions on how to perform a behavior (4.1); Anticipation of future reward (14.10); Identification of self as role model (13.1)	Teachers
	CATCH Coordination Toolkit	Teachers/ School staff	Modeling of behaviors (6.1); Goal setting (1.1); Review of outcome goals (1.7)	Teachers

*The Behavior Change Technique Taxonomy (v1) [74] was used to identify the behavior change techniques utilized in the interventions

Unique to the TGEG study is the collaboration between Texas A&M AgriLife Extension and the University of Texas School of Public Health. AgriLife Extension was established (Smith-Lever Act, 1914) to disseminate research from Land Grant Universities in the U.S. (created by the Morrill Act, 1862) to agricultural producers and their families. Cooperative Extension Services exist in all 50 states and provide an untapped resource for providing effective health interventions for families across the nation. By building upon the existing programs and volunteer networks provided by the Texas A&M AgriLife Extension Service, the TGEG study served as a demonstration of a novel and effective partnership which may provide a blueprint for effective replication nationally.

### Description and implementation of interventions

#### Coordinated School Health (CSH) program

As mentioned above, all schools participating in our study were required to have selected the CATCH program as their CSH program. CATCH (Coordinated Approach To Child Health) is a school-based health program designed to promote physical activity and healthy food choices and prevent tobacco use. CATCH transforms a child’s environment, culture, and society by coordinating child health efforts across all aspects of the educational experience: classroom, food services, physical education, and family [55, 56]. CATCH vocabulary (e.g., “Go, Slow, and Whoa!” foods) and philosophy were incorporated in both LGEG and WAT in order to integrate the interventions. At the beginning of the school year, all study schools were provided with the CATCH Coordination Kit and a training session on CATCH to ensure uniformity in delivery across schools. However, the study staff did not provide any additional assistance with the implementation of CATCH. Measurement of CATCH implementation fidelity was built into the process evaluation protocol.

#### The Learn, Grow, Eat & Go! (LGEG) Intervention

To create the LGEG intervention, a Junior Master Garden program, entitled the “Health and Nutrition from the Garden,” was modified significantly to include SCT-based strategies [54] targeting psychosocial variables and our key behavioral outcomes [57]. The new LGEG intervention includes school gardens for each participating classroom, a classroom curriculum, a student garden journal, and Family Story reading activities. Students grew vegetables throughout the year and participated in vegetable recipe demonstrations. Students also took home English and Spanish recipe cards (which featured kitchen math activities). Family Stories included reading assignments for students to complete at home with the parent/guardian. The stories paralleled the classroom curriculum, featured four of the vegetables, and used consistent messages to model small steps a family can take to be healthy. All lessons were aligned with the Texas Essential Knowledge and Skills (TEKS) as well as the State of Texas Assessments of Academic Readiness (STAAR) Performance Standards. For this study, LGEG was implemented throughout the school year in 3^rd^ grade classrooms.

At the beginning of the school year, all participating 3^rd^ grade teachers at LGEG or Combined schools attended a five-hour training session. This session included an overview of the intervention, activities to familiarize the teachers with intervention components, introduction to local Extension partners, the Teacher Intervention Activity Log and the research/data collection tools. The local AgriLife Extension educators, Master Volunteers and TGEG Project Specialists provided support for the garden installation, the vegetable tasting and conducted the related vegetable recipe demonstration for each classroom. Throughout the year, teachers worked with AgriLife Extension agents, Master Volunteers, and other volunteers to implement the LGEG intervention. The TGEG local Extension Project Specialists provided the coordination and technical assistance throughout the year. The process evaluation assessed each school’s implementation of the specific LGEG components.

#### The Walk Across Texas (WAT) intervention

WAT is a best-practice PA program developed by AgriLife Extension and includes multiple program components designed to establish the habit of regular PA [58]. For the TGEG study, components of the WAT program included a kick-off event, a classroom team competition to walk 832 miles per class in eight weeks, a home family bonus miles form, and an end-of-program celebration. In addition, each teacher was asked to perform two classroom activity break lesson plans during each week throughout the program. All lessons were aligned with the TEKS as well as the STAAR Performance Standards. Weekly English and Spanish newsletters featuring both healthy PA and eating tips were added to enhance family engagement through messaging and parent–child activities. Students also took home a Walking Bingo Activity Card to encourage family outdoor activities in their community.

At the beginning of the school year, all participating 3^rd^ grade teachers at WAT or Combined schools attended a three-hour training session. Each training session included an overview of the intervention, activities to familiarize the teachers with intervention components, an introduction to local Extension partners, the Teacher Intervention Activity Log and the research/data collection tools and plan. Throughout the year, either classroom teachers, Parent Support Specialists or PE teachers implemented the WAT intervention. The TGEG Project Specialist provided technical assistance throughout the year. Process evaluation assessed each school’s implementation of the specific WAT components.

### Recruitment of schools

The setting for this study included low-income elementary schools. Inclusion criteria of the schools included: 1) classified as a Title 1 (defined as schools with at least 40 % of student population living in low-income households); 2) located within one of the study’s geographical areas of Texas; 3) implementation of CATCH as a CSH program; 4) school commitment at the district, principal, and teacher level. Admission to individual public schools in the U.S. is usually based on residency. A large portion of school revenues come from local property taxes, and hence are dependent on how wealthy or poor these localities are. Thus, public schools vary widely in the resources they have available per student, resulting in large differences in school quality, class size, and curriculum from one district to another. These geographical differences are often compounded by residential segregation of minorities. Therefore, when conducting research studies in U.S. schools, it is important to have specific inclusion criteria for percent of children living in low-income households.

### Randomization of schools

For both years, the four schools in each geographic region or county site were randomized to treatment by the project PI listing the elementary school name on an index card & folding the card to conceal the school name. Treatments were then assigned through a blind drawing by a non-research staff member. The first school drawn was assigned to CATCH (control); second assigned to CATCH + LGEG; third assigned to CATCH + WAT; last drawn assigned to CATCH + WAT + LGEG. The school assignment was then communicated to the school district partner to inform the school principal who had previously committed by letter to implement the randomly assigned treatment.

### Recruitment of students and parents

The goal for this study was to recruit 50 student/parent dyads per school, from 32 schools, for a total of 1600 students. A priori power analysis calculations suggested that with a sample size of 1600, and a potential attrition rate of 40 %, the estimated power to detect a 5 % change in percent obese in any treatment group versus the control group is about 85 %, assuming alpha = 0.9, and an AR1 covariance structure between repeated measures, and a correlation of 0.9 across any two contiguous measures.

We recruited 3^rd^ grade students and their parents by sending TGEG Study Packets home from school to parents via the 3^rd^ grade students. Inclusion criteria for the students were: 1) enrollment in the 3^rd^ grade at a study school and 2) willingness to complete the Student Survey four times during the study. Exclusion criteria includes: 1) being on a special diet (i.e. a diet which would limit the consumption of certain foods due to medical or religious beliefs such as a ketogenic or gluten-free diet), and 2) primary language not English or Spanish. Inclusion criteria for the parents were: 1) ability to read in English or Spanish, and 2) parent/caretaker of a 3^rd^ grade child. The study packets contained a cover letter, active consent forms (both parent and child), a media release form (in case a child was featured in a picture to be posted on the TGEG website), and a Parent Survey. Parents could agree to let their child participate without participating themselves. Students received a small incentive at each data collection period (e.g. rulers, lunch bags, measuring spoons). Parents did not receive an incentive for participation. All recruitment and data collection procedures and protocols were approved by each university’s Institutional Review Board and by the appropriate school districts’ research authority.

### Data collection

Data for both outcome and process measures were collected from multiple sources (see Table 3). Self-report data from students were collected during the school day, requiring flexibility so that the protocol could be adapted to each school’s unique environment. Parents were asked to complete the Parent Survey at home and return the survey to the school via their student.

**Table 3:** Overview of TGEG Outcome and Process Measures

		Baseline	T2	T3	T4
Student Survey	Behavioral variables (V and SSB consumption and PA behaviors)	X	X	X	X
	Gardening experience	X	X	X	X
	Nutrition and Science Knowledge	X	X	X	X
	Psychosocial variables	X	X	X	X
Parent Survey	Behavioral variables (V and SSB consumption and PA behaviors) Gardening experience	X	X		X
	Psychosocial variables	X	X		X
	Child Health variables	X	X		X
	Program component experience (reach into home environment)		X		X
Stadiometer & Tanita Scales	Child BMI	X	X	X	X
Teacher questionnaires & implementation logs	Barriers and facilitators to Implementation	X	X		
	Perceived Implementation Success	X	X		
	Behavioral variables (V consumption and PA behaviors)	X	X		
	Food and PA environment in classroom	X	X		
	Assess program component implementation	X	X		
Principal interviews	Administrative support for intervention		X		
Extension Project Specialists Interviews	Intervention implementation fidelity	X			
Volunteers	Psychosocial variables (confidence, attitudes)	X	X		
	Behavioral Variables	X	X		
	Gardening Experience	X	X		

Abbreviations: *V* Vegetable, *SSB* Sugar-sweetened Beverage, *PA* Physical Activity

Process data were collected from teachers, principals, volunteers and AgriLife Extension Project Specialists. The 3^rd^ grade teachers provided information about program implementation related to the appropriate intervention components via a structured survey. They also provided information about their level of satisfaction with the intervention and about perceived changes in their own behaviors. School principals were interviewed by the TGEG evaluation team and were asked to provide information about administrative support for the intervention. Volunteers (Master Gardener, Master Wellness, parents) provided information about their self-efficacy for volunteering, health behaviors and gardening experience. AgriLife Extension project specialists scored classroom implementation of key program components for each teacher and school based on their in-class and in-school observations.

Researchers involved in the TGEG study were not blinded to the treatment assignment of the different schools. So although the TGEG Implementation Group and the TGEG Evaluation group involved different researchers, the study was not blinded.

### Description of measures

#### Student surveys

The key outcome variables for students included vegetable preference, exposure, and consumption, as well as consumption of SSB, and physical and sedentary activity behaviors. Items and scales included on the Student Survey were adapted from previously developed and validated instruments, including food intake questions targeting vegetables and SSB consumption from the School Physical Activity and Nutrition (SPAN) Survey [59–61], PA questions from the Marathon Kids Survey [62], and food preference questions from the GIMME5 Survey [63]. Other questions such as knowledge about gardening and frequency of family gardening activities were specifically developed for the TGEG Study. In terms of demographic data, students reported their gender and age. All items were translated into Spanish and back-translated into English. All questions were researched and developed by the research team, tested during the pilot study and refined for the full study [53]. Table 1 provides summary information for the measures included on the Student Survey.

#### Parent surveys

Parent self-report surveys included scales paralleling the Student Survey on consumption of vegetables and SSBs and engagement in PA, using items and scales adapted from other tested questionnaires or developed specifically for this study (Table 1). Parents were also asked to report on gardening experience and gardening with children (developed for this study), social support for their student’s healthy behaviors [63], cooking skills [64], and home availability of vegetables and SSBs [65]. Demographic items included questions such as gender, age, parent and student ethnicity/race, household characteristics, food security [66], and student health. Measures of parent-student interaction were primarily derived from the Parent Survey, and consisted mostly of two-item scales. The Gardening together variable was derived from the student survey. The Parent Survey was field tested with a small group of parents from the target population and revised slightly based on parental feedback. All items were translated into Spanish and back translated into English.

#### Student height and weight

Trained research staff used standard equipment (digital Tanita BWB 800S digital scale and PE-AIM-101 stadiometer) and calibration procedures to measure body weight to the nearest 0.1 kg and height to the nearest 1 mm as described in the National Center for Health Statistics. Child BMI [weight (kg)/stature (m)^2^] z-scores and percentiles for age and gender were computed using the 2000 CDC reference [67].

#### Teacher surveys

Self-report Teacher Surveys included questions on previous experience with healthy eating and PA school programs, school climate and barriers to implementation of interventions, usual healthy eating, PA and gardening behaviors and demographics such as number of years teaching, length of employment at school and district, gender, age, race/ethnicity and specific health education training. Teachers also completed a program implementation log tailored to the particular intervention that they were implementing. The logs provided dates and hours related to each intervention component completed by the teacher. Both instruments were developed by the research team and tested during the pilot study and refined for the full study.

#### School principal interviews

School principals were interviewed at the end of each intervention year by trained research staff. Interview questions included time in position at school and as an administrator, familiarity with health interventions, involvement of school staff and organizations such as PTA/PTO with intervention, perceived benefits and challenges to intervention, opinions and perceptions about the intervention’s effects on student involvement (or school/classroom engagement), behavior- and class-related outcomes, beliefs about parental involvement, potential for sustainability, overall recommendations to other school principals and ideas for improvements. The interview questions were tailored to the particular intervention being implemented at the school.

#### Volunteer surveys

The self-report Volunteer Surveys in English and Spanish included questions on past volunteer experience; volunteer self-efficacy related to implementing the intervention, TGEG training exposure; usual dietary, PA and gardening behaviors.

#### AgriLife extension project specialists implementation assessments

Project Specialists working with each school and each classroom completed a program implementation rating for each intervention by classroom. Based on standardized protocol, a number (1 for low, 2 for medium, and 3 for high) was assigned to each classroom.

### Data analysis

For the purpose of this overview paper, baseline data related to participant socio-demographic characteristics and the key outcome variables are presented (Table 4). Baseline distribution of socio-demographic characteristics at the household level (language at home, child participation in the Free and Reduced Price School Meals program, and food insecurity), parent level (gender, age, ethnicity of reporting parent), and child level (gender and age) across the four treatment conditions were cross-tabulated, and differences across treatment conditions were evaluated using chi-square statistics.

**Table 4:** Socio-demographic variables by treatment condition at baseline

	Comparison (%)	WAT (%)	LGEG (%)	WAT + LGEG (%)	Total (%)	*P*-value
Household Demographics (*n* = 1206)						
Language at home						
English	158(69.6)	238(77.5)	169(64.7)	255(75.8)	820(72.5)	
Spanish	68(29.9)	66(21.5)	91(34.8)	70(20.8)	295(26.0)	
Other	1(0.4)	3(0.9)	1(0.3)	11(3.2)	16(1.4)	
Total	227	307	261	336	1131	<0.001
Free/Reduced lunch						
No	41(17.9)	81(26.3)	56(21.4)	73(21.6)	251(22.1)	
Yes	188(82.1)	226(73.6)	205(78.5)	264(78.3)	883(77.8)	
Total	229	307	261	337	1134	0.13
Food insecurity						
Almost never/ never	133(57.3)	190(61.4)	147(55.6)	177(52.3)	647(56.6)	
Sometime	66(28.4)	73(23.6)	87(32.9)	116(34.3)	342(29.9)	
Almost always	33(14.2)	46(14.8)	30(11.3)	45(13.3)	154(13.4)	
Total	232	307	264	338	1143	0.08
Parent Demographics (*n* = 1206)						
Gender						
Male	18(7.7)	41(13.4)	27(10.3)	49(14.5)	135(11.8)	
Female	213(92.2)	265(86.6)	234(89.6)	289(85.5)	1001(88.1)	
Total	231	306	261	338	1136	0.07
Age						
Less than 25	3(1.4)	8(2.8)	3(1.2)	4(1.3)	18(1.7)	
25 to 29	42(19.9)	58(20.4)	51(21.2)	57(18.5)	208(19.9)	
30 to 34	71(33.6)	85(29.9)	85(35.4)	105(34.2)	346(33.2)	
35 to 39	44(20.8)	65(22.8)	45(18.7)	65(21.1)	219(21.0)	
40 to 44	30(14.2)	39(13.7)	33(13.7)	41(13.3)	143(13.7)	
45 to 49	12(5.6)	16(5.6)	13(5.4)	18(5.8)	59(5.6)	
50+	9(4.2)	13(4.5)	10(4.1)	17(5.5)	49(4.7)	
Total	211	284	240	307	1042	0.99
Ethnicity						
White	50(21.8)	52(18.8)	74(27.9)	86(25.3)	262(23.6)	
Black	50(21.8)	75(27.1)	22(8.3)	50(14.7)	197(17.7)	
Hispanic	123(53.7)	128(46.3)	154(58.1)	178(52.5)	583(52.5)	
Other	6(2.6)	21(7.6)	15(5.6)	25(7.3)	67(6.0)	
Total	229	276	265	339	1,109	<0.001
Student Demographics (*n* = 1326)						
Gender						
Boy	129(45.2)	173(51.4)	173(49.8)	177(49.5)	652(49.2)	
Girl	156(54.7)	163(48.5)	174(50.1)	180(50.4)	673(50.7)	
Total	285	336	347	357	1325	0.47
Age						
7- 8 years old	202(72.4)	243(72.5)	256(75.5)	222(62.7)	923(70.6)	
9 – 11 years old	77(27.6)	92(27.3)	83(24.4)	132(37.2)	384(29.3)	
Total	279	335	339	354	1307	0.02

Abbreviations: *LGEG* Learn, Grow, Eat & Go!, *WAT* Walk Across Texas

Hierarchical regression models with a logit link were used to estimate the prevalence of overweight and obesity under each of the four treatment conditions, and to evaluate if these prevalence statistics differed by treatment condition at baseline. Secondary outcome variables of interest for the TGEG evaluation included behavioral outcomes at the student and parent levels, as well as measures of student-parent interaction in the PA and healthy eating domains. Means of the key outcome variables for each treatment condition were estimated using hierarchical linear models, with school specified as a random intercept. Differences in mean values of these outcomes for each of the three intervention groups against the control group were evaluated for significance.

## Results

Although the original intent was to recruit a total of 32 schools in 4 counties, due to logistical issues, a total of 28 schools located in five different geographic areas in Texas participated in the study. Specifically, 16 schools (four per school district) in the 2012–2013 school year, and 12 schools (four per school district) in the 2013–2014 school year were randomly assigned to one of the four treatment groups. Of the 28 participating schools, eight were located in north central Texas, eight schools were on the southern coast, eight in east central and four in central Texas. The varied locations provided a diversity of cultures and growing conditions for the school gardens. All schools were classified Title I, with 85 % of the students across all schools eligible for the Free and Reduced Price School Meals program (range: 61 % – 99 %).

Participation rates varied by school with student participation ranging 24 % to 90 % of 3^rd^ graders per school, with a mean participation rate of 56 %. Our study goal of recruitment of 50 students per school was met in 56 % of the schools. In 64 % of the schools we were able to recruit at least 40 students. However, some of the participating schools had fewer than 50 eligible students per school and, therefore, it was impossible to reach our recruitment goal.

Sociodemographic data are presented for students (*n* = 1326), parents (*n* = 1206), and households (*n* = 1206) (Table 4). Across all treatment groups participants were ethnically diverse and low-income. Overall, 52 % of parents reported being Hispanic and 18 % African American; 26 % of parents reported speaking mostly Spanish at home. A high percentage of parents reported participation of their child in the Free and Reduced Price School Meals program, a commonly used proxy for poverty, which is substantially higher than the statewide participation rate of 60 % in 2012–13 [68]. In addition, 43 % of parents reported being sometimes or almost always food insecure. Table 4 also points to the presence of some sociodemographic differences across treatment conditions, particularly with regard to the distribution of ethnicity and language. Most of these differences derive from a larger proportion of Hispanic in the LGEG group. Overall, missing data for the sociodemographic variables was within a reasonable range. One exception is the large percent missing for the student race/ethnicity measure. Student race/ethnicity information was imputed from parent reports of race/ethnicity, but because not all parents chose to respond to the parent questionnaire, there is a larger than expected number of missing data for this variable.

Tables 5 and 6 present baseline data on key outcomes, including weight status prevalence among students, and distributions of secondary behavioral outcomes. At baseline, none of the three treatment conditions were significantly different from the control group in percent overweight or obese, or in percent obese. Percent overweight or obese across the four conditions varied from 46 % to 52.5 %, while percent obese ranged from 27 % to 37 %. In addition, none of the treatment groups differed significantly from the control group on any of the behavioral outcome variables (Table 6).

**Table 5:** Baseline key outcome variables by treatment condition, compared to Comparison group

Outcome	Comparison Mean (SE)	WAT Mean (SE) [p-value]*	LGEG Mean (SE) [p-value]*	WAT+ LGEG Mean (SE) [p-value]*
Student				
Percent overweight or obese	46.8 (3.1)	52.5 (2.7) [0.2]	49.2 (2.7) [0.6]	45.8 (2.7) [0.8]
Percent obese	31.1 (2.9)	36.5 (2.8) [0.2]	26.0 (2.0) [0.2]	26.7 (2.4) [0.2]
Vegetables preference	8.7 (0.2)	9.2 (0.2) [0.2]	9.1 (0.2) [0.2]	8.5 (0.2) [0.6]
Vegetable exposure	11.7 (0.5)	12.4 (0.5) [0.3]	12.1 (0.5) [0.6]	12.2 (0.5) [0.5]
Vegetable consumption	2.8 (0.2)	2.7 (0.2) [0.7]	2.6 (0.18) [0.5]	2.5 (0.17) [0.3]
SSB consumption	2.2 (0.2)	2.3 (0.2) [0.7]	2.1 (0.2) [0.8]	2.5 (0.2) [0.3]
MVPA	0.8 (0.0)	0.9 (0.0) [0.5]	0.9 (0.0) [0.1]	0.9 (0.0) [0.2]
Sedentary Behavior (spent more than 2 h in Sed. Beh.)	54.7 %	55.1 %	59.6 %	54.5 %
Parent				
Home availability of vegetables	8.8 (0.2)	8.6 (0.2) [0.4]	8.5 (0.2) [0.3]	8.7 (0.2) [0.5]
Home availability SSB	1.6 (0.1)	1.6 (0.1) [0.6]	1.7 (0.1) [0.3]	1.6 (0.1) [0.9]
Parental emotional support for increasing PA	15.1 (0.4)	15.1 (0.3) [0.9]	14.9 (0.4) [0.8]	15.2 (0.3) [0.8]
Parental support for decreasing sedentary behavior	6.3 (0.2)	6.4 (0.2) [0.9]	6.4 (0.2) [0.9]	6.4 (0.17) [0.9]
Student/parent Interaction (parent responses)				
Gardening together	2.0 (0.2)	2.0 (0.2) [0.9]	2.2 (0.2) [0.4]	2.2 (0.2) [0.6]
Eating meals together	2.8 (0.1)	2.7 (0.1) [0.6]	2.7 (0.1) [0.4]	2.8 (0.1) [0.8]
Engaging in PA together	3.9 (0.2)	3.9 (0.2) [0.8]	3.7 (0.2) [0.3]	3.9 (0.2) [0.7]
Preparing food together	1.2 (0.1)	1.3 (0.05) [0.1]	1.2 (0.1) [0.9]	1.3 (0.1) [0.4]

Abbreviations: *LGEG* Learn! Grow! Eat! Go!, *WAT* Walk Across Texas, *PA* physical activity, *SS B*sugar-sweetened beverages

*The p values were calculated for the comparison of the treatment group to the comparison group. No significant differences were found for any of the main outcome variables

**Table 6:** Student BMI

	Comparison (%)	WAT (%)	LGEG (%)	WAT + LGEG (%)	Total (%)	*P*-value
Normal Weight (<85^th^ percentile)	135 (53.2)	143 (47.5)	168 (50.8)	187 (54.2)	633 (51.4)	
Overweight (>= 85^th^ and <95^th^ percentile)	40 (15.8)	48 (15.9)	77 (23.3)	66 (19.1)	231 (18.8)	
Obese (> = 95^th^ percentile)	79 (31.1)	110 (36.5)	86 (25.9)	92 (26.7)	367 (29.8)	
Total	254	301	331	345	1231	<0.001

Abbreviations: *LGEG* Learn, Grow, Eat & Go!, *WAT* Walk Across Texas

Behavioral data reported by the students indicate moderate levels of exposure to and preference for vegetables. Over 90 % of students reported having been exposed to (ever tasted) corn, carrots and lettuce. Corn was also the most liked vegetable, followed by white potatoes. In terms of actual vegetable consumption, children reported eating vegetables about 2.6 times during the previous day. In addition, they reported consuming SSB 2.3 times the previous day. Eighty-eight percent of the students reported being moderately or vigorously active for 30 min the previous day while also reporting high levels of sedentary behavior (i.e. almost 4 h per day).

Parental instrumental support for healthy student behaviors was moderate to high at baseline. Both vegetables and SSB were reported by parents as being available at home “most of the time.” Parental support for increasing their student’s PA was relatively high, while their support for decreasing sedentary behaviors was moderate. Student-reported involvement in gardening activities along with parents was moderate. The extent to which students ate meals (breakfast, dinner) with family was relatively high in our baseline population.

## Discussion

Given past data indicating that lower income children are more likely to be overweight or obese [1, 69], the TGEG study targeted low-income schools in order to be able to study the intervention effects on students with the highest risk of obesity. The behavioral and BMI data collected at baseline indicate that the students and families targeted by the TGEG study were an appropriate priority population for obesity prevention efforts. Across the study groups, obesity rates ranged from 26 % to 36 %. In comparison, in 2011–2012, 18 % of U.S. children ages 6 to 11 were considered obese. Among Hispanic children, 26 % were classified as obese [1]. Thus, our participants were substantially more overweight and obese.

Baseline data on the student behavioral variables indicate low consumption of vegetables and high consumption of SSB, similar to other data describing high rates of SSB consumption among low-income populations [70]. Interestingly, both student-reported engagement in MVPA and sedentary behavior were relatively high. These health and behavioral findings are consistent with other studies that have targeted similar populations in Texas such as the CORD study [71], the TCOPPE study [72], and the SPAN study [73].

The demographic breakdown of the TGEG participants (mostly low-income and Hispanic) indicated the need for some special considerations related to data collection procedures and intervention materials. Specifically, the intervention materials needed to address specific socioeconomic, home environment, and cultural issues that could potentially influence dietary and PA behaviors of the participants. In addition to tailoring the interventions to our population, all study materials needed to be available in both English and Spanish and during data collection, there was a need for bilingual trained data collection staff.

In order to increase potential for adoption of the interventions upon completion of the study, the interventions were implemented using existing Texas AgriLife Extension resources. For example, county-based AgriLife Extension agents and trained Extension volunteers assisted with the building of the gardens and vegetable exposure activities. Trained AgriLife Extension educators and volunteers supported garden installation and maintenance and food exposures in participating school districts. Using the existing national Extension network increases the possibility of expanding implementation of the interventions across the state and nation.

While this study was ambitious, it has notable limitations. The nature of the intervention constrained us to randomize conditions at the school-level. With 28 schools, it was not possible to achieve perfect balance of all covariates across conditions. To limit the possibility of unobserved confounding resulting from such imbalance, we collected data on a large variety of parent and student covariates. Another limitation is the young age of the study population. The availability and scope of empirically tested measures suitable for the reading and comprehension skills of third-grade children is low; hence, we were limited to using very simple measures of behaviors to ensure validity in response. However, BMI, our primary outcome, is objectively measured. Related to the young age of our target population is the limitation that our evaluation measures did not include all the relevant SCT constructs, even though SCT was used to develop the strategies included in the interventions. Although we measured some SCT constructs (please note that in this paper we only mention our main outcome variables; additional variables were included in the instruments), because of the need to limit the number of items on the surveys, we were unable to include all SCT constructs which were targeted through the strategies. Despite these limitations, this is an important study, examining the impact of scalable school-based programs on healthy eating and PA on children at an age when such programs are acceptable and feasible. The study’s design, a factorial group RCT with 4 different groups, enhances the internal validity of the study. In addition, the relatively large and diverse sample will contribute to the generalizability of the results of this study. Lastly, the inclusion of existing networks for implementation will enhance the potential of dissemination of the TGEG interventions.

## Conclusions

The TGEG study is an on-going study assessing the independent and combined impact of gardening, nutrition and PA intervention(s) on the prevalence of healthy eating and PA behaviors and weight status among low-income 3rd grade students and parents. Compared to other school-based interventions, the TGEG study is distinguished by its factorial RCT study design, its large sample size, the high percentage of Hispanic participants and low-socio-economic status study population (a population at particular high risk for obesity), its inclusion of both nutrition and PA as targeted behaviors, and its ecological approach to changing the school environment to support healthy outcomes. Additional data collections have been completed and are being analyzed on dimensions related to program implementation variation. Findings to date relate to the feasibility and challenges of the intervention, as well as provide information on the demographics, diet and activity behaviors, and weight status of 3^rd^ grade children from an ethnically diverse, low-income population.

## Abbreviations

CATCH, Coordinated Approach to Child Health; CSH, Coordinated School Health; F&V, Fruit and vegetables; LGEG, Learn, Grow, Eat & Go!; PA, Physical Activity; RCT, Randomized Control Trial; SCT, Social Cognitive Theory; SSB, Sugar Sweetened Beverages; TGEG, Texas Grow Eat Go!; WAT, Walk across Texas.
